# Bridging biodiversity gaps: Updated taxonomy and diversity of Sri Lankan *Myllocerus* (Coleoptera, Curculionidae, Entiminae)

**DOI:** 10.3897/zookeys.1284.191034

**Published:** 2026-07-03

**Authors:** Dilshara D. Wijesinghe, Ki-Jeong Hong

**Affiliations:** 1 Department of Plant Medicine, Sunchon National University, Suncheon 57922, Republic of Korea Department of Plant Medicine, Sunchon National University Suncheon Republic of Korea

**Keywords:** Biodiversity, Curculionidae, Entiminae, identification key, Cyphicerini, Sri Lanka

## Abstract

The genus *Myllocerus* includes numerous economically important species because both adults and larvae cause damage to agricultural, horticultural, and forest crops. To date, 11 species of *Myllocerus* have been recorded from Sri Lanka. However, taxonomic knowledge remains limited, and specimens in established Sri Lankan institutional collections are scarce, with most historical material housed in overseas institutions. This study presents an updated account of *Myllocerus* species in Sri Lanka, including diagnostic characters, habitus images, distributional data, and the first illustrated identification key. *Myllocerus angulatipes*, *M. equinus*, and *M. zeylanicus* are Sri Lankan endemics, while *M. undecimpustulatus undatus* is native to Sri Lanka and adventive in the USA. Compared with southern India, the known Sri Lankan diversity of *Myllocerus* is low. This highlights the need for further field study and taxonomic revision.

## Introduction

Weevils (Coleoptera: Curculionidae) are a hyperdiverse family of beetles, within which the largest subfamily is Entiminae Schönherr, 1823 (Oberprieler et al. 2007). Entiminae are anatomically characterized by a short rostrum with adelognathous mouthparts, in which the maxillae are concealed by the prementum; mandibles bear deciduous cusps that facilitate the emergence of teneral adults from earthen pupal cells (Oberprieler et al. 2007). Some entimines, e.g. *Calomycterus setarius* Roelofs, 1873, and *Myllocerus undecimpustulatus* Faust, 1891 cause serious damage to crops (Hunt et al. 2003; O’Brien et al. 2006).

The genus *Myllocerus* Schönherr, 1823 belongs to the subtribe Myllocerina Pierce, 1913, tribe Cyphicerini Lacordaire, 1863, under subfamily Entiminae Schönherr, 1823 (Alonso-Zarazaga et al. 2023). *Myllocerus* Schönherr, 1823 is distinguished by the rostrum being continuous with the head and symmetrically emarginated at the apex, epistome bounded by separate carinae, subdorsal scrobes, antennae with a scape extending beyond the anterior margin of the prothorax, procoxae nearly at the center of the prosternum, dentate femora, and free claws (Ramamurthy and Ghai 1988). Many species are essentially defoliators in their adult phase and root feeders in their larval stages (Nagaraja 2019). Both adults and larvae are economically important, as the adults cause severe defoliation in many agricultural, horticultural, and forest crops, while the larvae feed on rootlets in the soil (Nagaraja 2019).

*Myllocerus* is predominantly distributed in the tropical zone and is known from the Palaearctic, Indomalayan, Afrotropical, and Australasian regions (Ramamurthy and Ghai 1988). There are 340 species recorded worldwide (Yunakov et al. 2026); 11 species are distributed in Sri Lanka (Wijesinghe and Hong 2025). From 1775 to 1977, more than two-thirds of the known *Myllocerus* species were described by various coleopterists, among whom Arthur Mills Lea, Guy Anstruther Knox Marshall, Eduard Voss, and Alphonse Hustache were particularly significant contributors. In neighboring India, 90 species have been recorded, of which 42 occur in southern India, a region geographically close to Sri Lanka (Marshall 1916; Ramamurthy and Ghai 1988; Nagaraja 2019); notably, six species are shared between India and Sri Lanka.

Several studies have reported the pest status of *M. curvicornis* (Fabricius, 1792), *M. discolor* Boheman, 1834, *M. undecimpustulatus undatus* Marshall, 1916, *M. subfasciatus* Guérin-Méneville, 1843, and *M. viridanus* (Fabricius, 1775) that damage various crops in Sri Lanka (Marshall 1916; Butani 1979; Shanthichandra et al. 1990; Neal 2013; Shanmugam et al. 2018; Rajan and Ghosh 2019; Wijerathna et al. 2024). However, no taxonomic work has been carried out since Ramamurthy and Ghai (1988). We present an updated and extended review with a key to Sri Lankan species and habitus images to facilitate further research on *Myllocerus* diversity in the region. This study aims to facilitate identification of *Myllocerus* species by users ranging from farmers to taxonomists of Sri Lanka.

## Materials and methods

A list of the genus *Myllocerus* recorded from Sri Lanka based on literature records was obtained from previously published articles (Ramamurthy and Ghai 1988; Wijesinghe and Hong 2025). Synonyms are presented as literature records and are based on published taxonomic catalogues and revisions (Marshall 1916; Ramamurthy and Ghai 1988; Alonso-Zarazaga et al. 2023; Wijesinghe and Hong 2025). Individual references cited within synonyms are treated as literature records and are not all repeated in the reference list; instead, the principal sources used are listed in the references section.

The distribution of *Myllocerus* species within the country is primarily based on known localities from the weevil checklist by Wijesinghe and Hong (2025) and integrated with confirmed observations from iNaturalist (https://www.inaturalist.org) and GBIF.org (2025) (https://www.gbif.org). To ensure data reliability, only GBIF records based on preserved specimens were included, while records categorized as human observations were excluded. iNaturalist records were treated independently and manually verified based on image quality, agreement among identifiers, and consistency with published species distributions; records lacking sufficient diagnostic evidence were excluded. We summarized distributional information and references in Table 1. Geographic locality coordinates compiled from published sources and field collections are provided in Suppl. material 1. Host plant families, associated taxa, and the number of associated species of *Myllocerus* are summarized in Table 2. All host plants are presented using scientific names, along with their respective families, to ensure consistency and clarity across regions.

**Table 1.** *Myllocerus* Schönherr, 1823 (Curculionoidea: Entiminae) species recorded for Sri Lanka and their known distributions in the country.

**Taxon**	**Distribution in Sri Lanka**	**References**
*M. angulatipes* Marshall, 1916	Dikoya	Marshall (1916: 316)
*M. curvicornis* (Fabricius, 1792)	Batticaloa, Colombo, Galkiriyagama*, Bakamuna*, Habarana, Haragama, Kandy, Kantalai, Kekirawa, Kitulgalle, Nalanda, Negombo, Neligama, Oddnechuddan, Puttalam, Rajakadaluwa, Teldeniya, Trincomalee, Weligama	Heller (1901: 338); Marshall (1916: 343); Voss (1957: 102)
*M. delicatulus* Boheman, 1842	Negombo, Anuradhapura, Hettipola, Puttalam, Kuchchaveli, Mihintale, Dambulla, Kandy, Teldeniya, Alut Oya, Haldumulle, Halumpahani, Madulsima, Ragala	Marshall (1916: 304); Voss (1957: 101); Ramamurthy and Ghai (1988: 456)
*M. dentifer* (Fabricius, 1792)	Galle, Colombo, Haragama, Kantalai, Uva	Marshall (1916: 347); Voss (1957: 102); iNaturalist (2025)
*M. discolor* Boheman, 1834	Nalanda, Eppewala, Batticaloa, Haragama, Kantalai, Uva, Ampara, Kurunegala	Heller (1901: 338); Marshall (1916: 349, 350); Voss (1957: 102); iNaturalist (2025)
*M. equinus* Ramamurthy & Ghai, 1988	No known localities	Ramamurthy and Ghai (1988: 464)
*M. fringilla* Faust, 1897	Puttalam, Trincomalee	Heller (1901: 338)
*M. subfasciatus* Guérin-Méneville, 1843	Anuradhapura, Haputala, Nalanda, Dikoya, Bogawantalawa, Balangoda, Peradeniya, Kandy, Weliweriya*	Heller (1901: 338); Marshall (1916: 346); Voss (1957: 102)
*M. undecimpustulatus undatus* Marshall, 1916	Weligama, Kandy, Kurunegala, Puttalam, Anuradhapura, Polonnaruwa, Haragama, Kantalai, Kalutara, Ratmalana, Mannar, Dankotuwa, Dambulla, Buttala, Divulapitiya	Marshall (1916: 350); Voss (1957: 102); iNaturalist (2025)
*M. viridanus* (Fabricius, 1775)	Colombo, Negombo, Galle, Balaugoda, Dikoya, Kandy, Elephant Pass, Manner, Nugawela	Heller (1901: 338); Marshall (1916: 301); Voss (1957: 101)
*M. zeylanicus* Marshall, 1916	Kandy, Balangoda, Kitulgalle, Teldeniya	Marshall (1916: 315); Voss (1957: 101)

*Locations of Sri Lanka where authors collected *Myllocerus* Schönherr, 1823 species during this study.

**Table 2.** Host plants and associated species of the genus *Myllocerus* Schönherr, 1823.

**Family**	**Host plants**	**No. of plants**	**No. of species**
Anacardiaceae	*Mangifera indica*	1	4
Apiaceae	*Daucus carota*	1	1
Arecaceae	*Cocos nucifera*, *Phoenix roebelenii*	2	2
Asteraceae	*Helianthus annuus*	1	2
Caricaceae	*Carica papaya*	1	1
Chrysobalanaceae	*Chrysobalanus icaco*	1	1
Combretaceae	*Conocarpus erectus*	1	1
Convolvulaceae	*Ipomoea batatas*	1	3
Cucurbitaceae	*Trichosanthes cucumerina*, *Cucurbita* spp., *Luffa acutangula*	3	3
Euphorbiaceae	*Ricinus communis*, *Acalypha* spp.	2	1
Fabaceae	*Arachis hypogaea*, *Cajanus cajan*, *Glycine max*, *Vigna mungo*, *Vigna unguiculata*, *Vigna aconitifolia*, *Lablab purpureus*, *Psophocarpus tetragonolobus*, *Medicago sativa*, *Trifolium alexandrinum*, *Cyamopsis tetragonoloba*, *Crotalaria juncea*, *Indigofera* spp., *Sesbania bispinosa*, *Sesbania* spp., *Dalbergia sissoo*, *Dalbergia paniculata*, *Erythrina lithosperma*, *Gliricidia sepium*, *Vachellia nilotica*, *Acacia decurrens*	21	6
Lamiaceae	*Tectona grandis*	1	2
Linaceae	*Linum usitatissimum*	1	1
Lythraceae	*Punica granatum*, *Lagerstroemia indica*	2	1
Malvaceae	*Gossypium* spp., *Corchorus* spp., *Abelmoschus esculentus*, *Theobroma cacao*, *Alcea rosea*	5	6
Melastomataceae	*Melastoma malabathricum*	1	1
Moraceae	*Ficus carica*, *Morus* spp.	2	3
Moringaceae	*Moringa oleifera*	1	1
Musaceae	*Musa* spp.	1	1
Muntingiaceae	*Muntingia calabura*	1	1
Myrtaceae	*Psidium guajava*, *Eucalyptus* spp.	2	2
Plumbaginaceae	*Plumbago zeylanica*	1	1
Poaceae	*Oryza sativa*, *Zea mays*, *Triticum aestivum*, *Sorghum bicolor*, *Pennisetum glaucum*, *Eleusine coracana*, *Setaria italica*, *Imperata cylindrica*, *Saccharum officinarum*	9	4
Rhamnaceae	*Ziziphus mauritiana*, *Ziziphus jujuba*	2	3
Rosaceae	*Prunus persica*, *Prunus* spp., *Malus domestica*, *Fragaria × ananassa*, *Rosa* spp., *Eriobotrya japonica*, *Prunus dulcis*	7	6
Rutaceae	*Citrus limon*, *Aegle marmelos*	2	3
Sapindaceae	*Litchi chinensis*	1	3
Sapotaceae	*Manilkara zapota*	1	1
Solanaceae	*Solanum melongena*, *Solanum tuberosum*, *Capsicum* spp.	3	3
Theaceae	*Camellia sinensis*	1	2
Urticaceae	*Boehmeria nivea*	1	1
Verbenaceae	*Lantana camara*	1	2

The majority of specimens examined and illustrated in this study are deposited in the Natural History Museum, London (**NHMUK**). The specimens collected by authors are deposited in the Sunchon National University, Suncheon, South Korea (**SCNU**). Specimens were identified based on external anatomical characters using original species descriptions, available taxonomic keys, and relevant revisions of *Myllocerus* (Faust 1897; Marshall 1916; Voss 1957; Ramamurthy and Ghai 1988; Nagaraja 2019). Identifications were verified through direct comparison with reference specimens from NHMUK. Images were captured using a Leica M125 stereo microscope (Leica Microsystems, Wetzlar, Germany) equipped with a Dhyana 400DC CMOS digital camera and processed using Mosaic v. 2.4 stacking software (Mosaic2.4.exe; v. 2.4.9.0; http://www.tucsen.com) and edited in Adobe Photoshop (v. 27, Adobe Inc., San Jose, California, USA). An identification key to species of the genus *Myllocerus* of Sri Lanka was developed based on detailed anatomical examination and previously published diagnostic data.

## Taxonomic account

### Subfamily Entiminae Schönherr, 1823

**Tribe Cyphicerini Lacordaire, 1863**

#### Subtribe Myllocerina

Taxon classificationAnimaliaColeopteraCurculionidae

Pierce, 1913

2AB6F261-2643-5B55-A470-51547AD055E8

##### Diagnosis.

Prothorax in lateral view with anterior margin straight and lacking vibrissae.

### Identification key to genera of the subtribe Myllocerina in Sri Lanka

**Table:** 

1	Metatibiae finely serrate externally, mesotibiae strongly curved	***Epicalus* Motschulsky, 1858**
–	Tibiae not serrate externally, mesotibiae not curved. Rostrum continuous with the head	**2**
2	Distinct elytral humeri, metacoxae not reaching elytral margin. Femora always with at least one tooth (teeth can be two or three). Distal margin of rostrum symmetrically emarginated, epistome bounded by posterior carina	***Myllocerus* Schönherr, 1823**
–	No elytral humeri, sides rounded, metacoxae reaching elytral margin. Epistome extending posteriorly beyond middle of pterygia. Metanepisternal suture more or less obliterate. Intercoxal process on the anterior margin of abdominal ventrite 1 rounded or angulate, narrower than the metacoxae	***Ptochus* Schönherr, 1823**

#### Genus Myllocerus

Taxon classificationAnimaliaColeopteraCurculionidae

Schönherr, 1823

3288CA03-A19D-5DB3-9C24-871B06586A6B

Myllocerus Schönherr, 1823: 1144. [Type species: Curculio curvicornis Fabricius, 1792, by original designation].
= Macrocorynus Schönherr, 1823: 1144. [Type species: Curculio discoideus Olivier, 1807].
= Epherina Pascoe, 1869: 102. [Type species: Epherina longicornis Pascoe, 1869].
= Idaspora Pascoe, 1869: 101. [Type species: Idaspora terrea Pascoe, 1869].
= Proxyrus Pascoe, 1870: 437. [Type species: Proxyrus abstersus Pascoe, 1870].
= Hyperstylus Roelofs, 1873: 171. [Type species: Hyperstylus pallipes Roelofs, 1873].
= Proxyrodes Blackburn, 1892: 48. [Type species: Proxyrodes maculatus Blackburn, 1892].
= Exmyllocerus Voss, 1937: 230. [Type species: Myllocerus ginfushanensis Voss, 1933].
= Hypomyllocerus Voss, 1937: 231. [Type species: Myllocerus inflatus Voss, 1937].
= Pachymyllocerus Voss, 1937: 235. [Type species: Myllocerus durus Voss, 1937].
= Isomyllocerus Marshall, 1954: 214. [Type species: Curculio dorsatus Fabricius, 1798].
= Pseudocanoixus Voss, 1958: 23. [Type species: Macrocorynus fallaciosus Voss, 1958].
= Calomyllocerus Voss, 1959: 408. [Type species: Myllocerus liesenfeldti Voss, 1959].
= Mylloceroversus Hoffmann, 1961: 644. [Type species: Myllocerus acaciae Hoffmann, 1961].

##### Diagnosis.

General color varies to a great extent, ranging from pale white to bright metallic with stripes, blotches, and spots of various patterns. Body elongate, more than twice as long as broad; sometimes cylindrical, more commonly oblong to ovate; convex dorsally, flat ventrally. Head wider than long; eyes dorsal to sublateral, variable in convexity. Rostrum stout, variable in shape; epistomal emargination ranging from deep and triangular to shallow and rounded; epistome bounded posteriorly by distinct carina. Scrobes dorso-lateral, visible from above, very deep anteriorly, abruptly shallow posteriorly, curved downwards and fading anteriorly of eye; genae dilated or not. Rostral carina transverse or longitudinal, distinct up to base or fading before reaching it. Antennae inserted near apex of rostrum; scape elongate, curved, extending beyond anterior margin of prothorax; funicle 7-jointed, funicular antennomeres 1 and 2 longer than others; club compact, spindle-shaped, 4-jointed. Prothorax subcylindrical to subtrapezoid; with posterior margin truncate or bisinuate, without ocular lobe or vibrissae on anterolateral margin. Elytra oblong, distinctly broader than prothorax at elytral humeri, distinct elytral humeri with ten punctate elytral striae; sides generally subparallel from elytral humeri to beyond mid-length, margins gently sinuate above metacoxa. Legs with procoxa contiguous, metacoxa not reaching margin of elytra; femora clavate and dentate (with 1–3 teeth), always on distal half; tibiae simple or occasionally basally sinuate, protibiae sometimes internally angulate or bisinuate; metatibial corbels absent; tarsi slender, tarsomere 1 elongate, 3 broadly ovate and bilobed; claws free. Abdominal ventrite 1 with intercoxal process ogival and narrower than metacoxa; abdominal ventrite 2 as long as or longer than 3 and 4 combined, separated from ventrite 1 by a curved incision which is shallower medially.

##### Distribution.

Afrotropical and Oriental Regions, southern and eastern Palaearctic and Australia (Alonso-Zarazaga and Lyal 1999; Alonso-Zarazaga et al. 2023); one species is adventive in the Nearctic Region (Hunsberger 2003).

### Identification Key to Sri Lankan species of the genus *Myllocerus* Schönherr, 1823

Fig. 1

**Figure 1.**
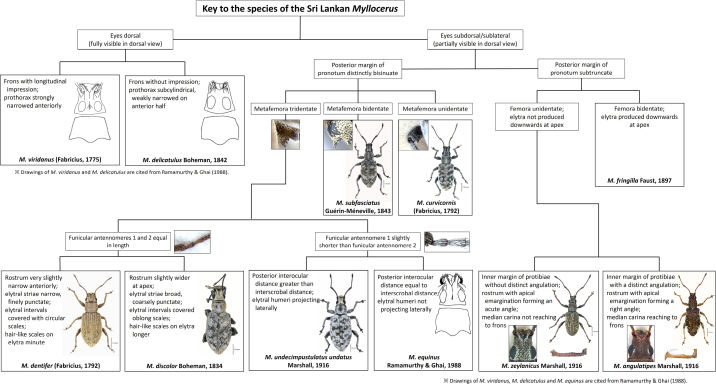
Illustrated key to Sri Lankan species of the genus *Myllocerus* Schönherr, 1823.

**Table:** 

1	Eyes dorsal	**2**
–	Eyes subdorsal/ sublateral	**3**
2	Prothorax strongly narrowed anteriorly; Frons with longitudinal impression	***M. viridanus* (Fabricius, 1775)**
–	Prothorax subcylindrical with rounded sides, weakly narrowed on anterior half; Frons without impressions	***M. delicatulus* Boheman, 1842**
3	Posterior margin of pronotum distinctly bisinuate	**4**
–	Posterior margin of pronotum subtruncate	**9**
4	Metafemora unidentate	***M. curvicornis* (Fabricius, 1792)**
–	Metafemora bidentate or tridentate	**5**
5	Metafemora bidentate	***M. subfasciatus* Guérin-Méneville, 1843**
–	Metafemora tridentate	**6**
6	Funicular antennomeres 1 and 2 equal in length	**7**
–	Funicular antennomere 1 slightly shorter than funicular antennomere 2	**8**
7	Rostrum very slightly narrowing from base to apex; elytral striae narrow and finely punctate; elytral vestiture by circular scales	***M. dentifer* (Fabricius, 1792)**
–	Rostrum slightly wider at apex; elytral striae broad and coarsely punctate; elytral vestiture by oblong ovate scales	***M. discolor* Boheman, 1834**
8	Posterior interocular distance greater than interscrobal distance; elytral humeri projecting laterally	***M. undecimpustulatus undatus* Marshall, 1916**
–	Posterior interocular distance equal to interscrobal distance; elytral humeri not projecting laterally	***M. equinus* Ramamurthy & Ghai, 1988**
9	Femora bidentate; elytra produced downwards at apex	***M. fringilla* Faust, 1897**
–	Femora unidentate; elytra not produced downwards at apex	**10**
10	Inner margin of protibiae without distinct angulation; rostrum with apical emargination forming an acute angle; median carina not reaching to frons	***M. zeylanicus* Marshall, 1916**
–	Inner margin of protibiae with a distinct angulation; rostrum with apical emargination forming a right angle; median carina reaching to frons	***M. angulatipes* Marshall, 1916**

#### Myllocerus angulatipes

Taxon classificationAnimaliaColeopteraCurculionidae

Marshall, 1916

30F4CA72-2B9A-58F0-920E-1039810404B2

Fig. 2A, B

Myllocerus angulatipes Marshall, 1916: 316. Type locality: Ceylon-Dikoya; Type repository: NHMUK. Type examined.

##### Material examined.

***Paratype***. Sri Lanka • 1♀; Central Province, Dikoya, 3800–4200 ft; 6 Dec. 1881–16 Jan. 1882; G. Lewis leg.; NHMUK, NHMUK013663874.

**Figure 2.**
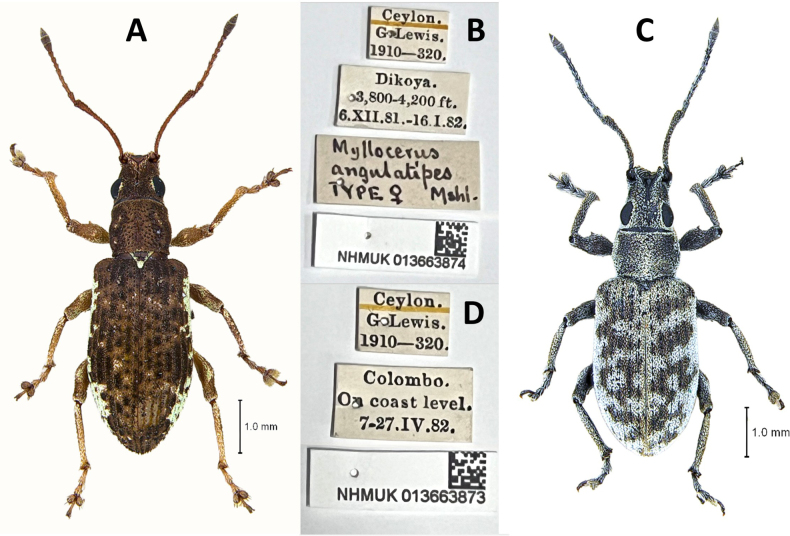
Sri Lankan *Myllocerus* Schönherr, 1823 species examined from the National History Museum, London. **A, B**. *Myllocerus angulatipes* Marshall, 1916; **A**. Habitus; **B**. Labels; **C, D**. *Myllocerus curvicornis* (Fabricius, 1792); **C**. Habitus; **D**. Labels.

##### Diagnosis.

Body dark brown; dorsal surface with brown scaling, variegated with darker patches on the elytra. Head bearing a broad lateral green stripe ventral to eye, continued along prothorax and elytra; on elytra stripe broader and extending to elytral stria 6 near base and to elytral stria 5 posterior to mid-length, irregular and variable. Head finely striolate, the striolation visible through thin scaling; eyes lateral and convex; interocular distance broader than length of eye. Rostrum about as long as broad in ♂ and slightly shorter in ♀, narrowed from base to beyond mid-length; apical emargination moderately deep, forming a right angle. Antennae reddish brown; scape with sparse minute pale scales and curved black setae; funicular antennomeres 1 and 2 equal, funicular antennomere 3 longer than 4; funicular antennomere 7 longer than broad. Prothorax transverse; sides rounded and broadest at mid-length; shallowly constricted and transversely impressed anteriorly and posteriorly; posterior margin truncate and not broader than anterior margin, which is rounded dorsally. Elytra subtruncate at base, parallel-sided in ♂ and slightly dilated posteriorly in ♀; dorsal surface almost flat; disc with a small transverse flattened area posterior to scutellar shield, followed posteriorly by a shallow, broad, oblique impression extending from elytra humeri to suture. Legs reddish brown; femora with a single tooth; inner margin of protibiae with a distinct angulation proximal to mid-length, weaker on metathoracic pair.

##### Remarks.

This species is similar to *M. zeylanicus* Marshall, 1916, but differs by the following characters: head bearing a broad lateral green stripe ventral to eye, continued along prothorax and elytra; rostrum with apical emargination forming a right angle, median carina reaching to frons; inner margin of protibiae with a distinct angulation proximal to mid-length.

##### Plant association.

Unknown.

##### Distribution.

Sri Lanka.

#### Myllocerus curvicornis

Taxon classificationAnimaliaColeopteraCurculionidae

(Fabricius, 1792)

1EC4932D-5545-5163-AC59-46A7F9C76352

Fig. 2C, D

Curculio curvicornis Fabricius, 1792: 488. Type locality: India orientalis; Type repository: Zoological Museum, University of Copenhagen. Type not examined.
= Myllocerus transmarinus Boheman, 1834: 429; Marshall 1916: 343; Ramamurthy and Ghai 1988: 455. Type locality: India orientalis; Type repository: Swedish Museum of Natural History, Stockholm.

##### Material examined.

Sri Lanka • 1♂ 1♀; North Central Province, Dambewatana, Galkiriyagama; 7°56.2'N, 80°33.61'E; 8 Jan. 2020; KJ. Hong leg.; on *Mangifera indica* (Anacardiaceae); SCNU • 1♂ 1♀; North Central Province, Bakamuna, Sirikanduyaya; 7°46.99'N, 80°50.54'E; 20 Feb. 2024; DD. Wijesinghe leg.; on *Gliricidia sepium* (Fabaceae); SCNU • 1♂; Western Province, Colombo; 7–27 Apr.1882; G. Lewis leg.; NHMUK, NHMUK013663873.

##### Diagnosis.

Body black to dark brown, with grey and brown scaling. Prothorax dorsally either dark brown with a narrow median pale brown line, or pale brown with a dark brown, longitudinal, lateral stripes. Elytra dark to pale brown dorsally, irregularly mottled with small grey spots, occasionally partly confluent. Lateral margins of head, prothorax, and elytra, as well as ventral surfaces, grey to whitish. Head with sparse obscure punctation concealed by scaling; eyes sublateral. Antennae dark brown, with depressed grey setae; funicular antennomere 2 distinctly longer than 1, funicular antennomere 3 slightly longer than 4. Prothorax transverse, sides slightly rounded, posterior margin moderately bisinuate, not broader than anterior margin. Elytra rather broad, separately rounded at base, deeply puncto-striate, punctures clearly visible through scaling; setae very short and recumbent. Legs dark brown with grey scaling; femora unidentate; tibiae simple.

##### Remarks.

This species is closely related to *M. subfasciatus* Guérin-Méneville, 1843, but differs by the following characters: funicular antennomere 2 distinctly longer than 1, funicular antennomere 3 slightly longer than 4; funicular antennomeres 2–6 without conspicuous white setae; metafemora unidentate.

##### Plant association.

*Psophocarpus tetragonolobus* (Fabaceae), *Vachellia nilotica* (= *Acacia arabica*) (Fabaceae), *Acacia decurrens* (Fabaceae), *Melastoma malabathricum* (Melastomataceae), *Theobroma cacao* (Malvaceae), *Camellia sinensis* (Theaceae), *Cocos nucifera* (Arecaceae), *Ziziphus mauritiana* (Rhamnaceae), *Rosa* spp. (Rosaceae), *Erythrina lithosperma* (Fabaceae), *Gliricidia sepium* (Fabaceae), *Mangifera indica* (Anacardiaceae) (Ramamurthy and Ghai 1988; Shanthichandra et al. 1990; Marshall 1916).

##### Distribution.

Sri Lanka, India.

#### Myllocerus delicatulus

Taxon classificationAnimaliaColeopteraCurculionidae

Boheman, 1842

60E76275-F978-5730-BF76-BF46F2D95916

Myllocerus delicatulus Boheman, 1842: 6. Type locality: Pondichery, India orientalis; Type repository: Swedish Museum of Natural History, Stockholm. Type not examined.
= Phyllobius mimicus Walker, 1859: 263; Marshall 1916: 304; Ramamurthy and Ghai 1988: 419. Type locality: Ceylon; Type repository: NHMUK.

##### Material examined.

No specimens examined; data based on published records.

##### Diagnosis.

Body dark brown, uniformly clothed with light yellowish green scaling. Head subconical; eyes dorsal, widely separated, interocular distance nearly equal to width of eye; frons without median impression. Rostrum as long as head; as long as or slightly broader than long, gradually narrowed anteriorly, sides straight; scrobes entirely dorsal, interscrobal distance slightly broader than interocular distance. Antennae black to dark brown; funicular antennomeres 1 and 2 elongate and subequal; funicular antennomere 3 slightly longer than 4; funicular antennomere 4 longer than 5; funicular antennomere 5 yellowish. Prothorax transverse, subcylindrical, sides parallel posteriorly and slightly narrowed only on anterior half; posterior sinuation shallow. Elytra with bases weakly rounded; strial punctures scarcely visible through scaling; elytral interstriae with scales circular, convex, disc-shaped. Legs yellowish; femora with a single small tooth (Marshall 1916).

##### Remarks.

This species can be distinguished from *M. viridanus* (Fabricius, 1775) by the following characters: head subconical, eyes more widely separated; interocular distance nearly as broad as width of eye; frons not impressed; antennae yellowish; prothorax weakly narrowed anteriorly; elytra with base distinctly rounded. It is also closely related to *M. thompsoni* Ramamurthy & Ghai, 1988, but differs by the following characters: interocular distance nearly as broad as width of eye; presence of a longitudinal ridge around eyes; funicular antennomeres 1 and 2 subequal; funicular antennomeres 3–6 not equal, progressively elongate; and elytral interstriae with scales distinctly smaller (Ramamurthy and Ghai 1988).

##### Plant association.

*Morus* spp. (Moraceae), *Litchi chinensis* (Sapindaceae), *Lantana camara* (Verbenaceae) (Ramamurthy and Ghai 1988).

##### Distribution.

Sri Lanka, India.

#### Myllocerus dentifer

Taxon classificationAnimaliaColeopteraCurculionidae

(Fabricius, 1792)

18B846E1-FCEC-55A0-B60D-E93D52AAA535

Fig. 3A, B

Curculio dentifer Fabricius, 1792: 488. Type locality: India orientalis; Type repository: Zoological Museum, University of Copenhagen. Type not examined.

##### Material examined.

Sri Lanka • 1♀, Southern Province, Galle; 27 Nov.–4 Dec. 1881; G. Lewis leg.; NHMUK, NHMUK013663882.

**Figure 3.**
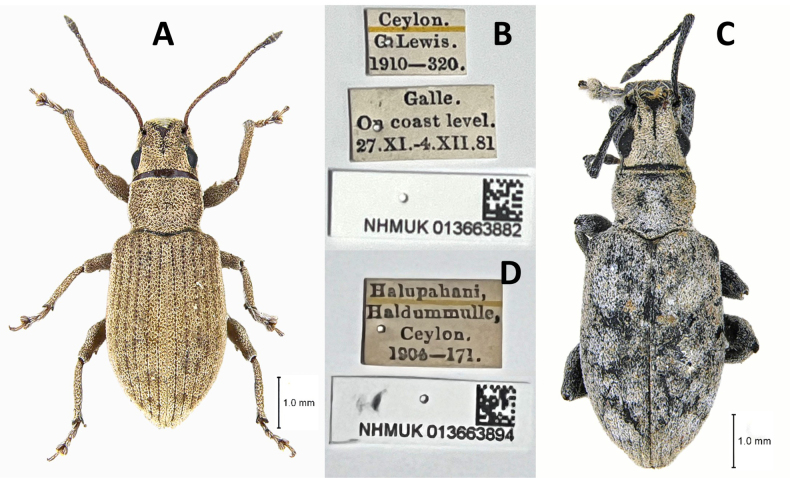
Sri Lankan *Myllocerus* Schönherr, 1823 species examined from National History Museum, London. **A, B**. *Myllocerus dentifer* (Fabricius, 1792); **A**. Habitus; **B**. Labels; **C, D**. *Myllocerus discolor* Boheman, 1834; **C**. Habitus; **D**. Labels.

##### Diagnosis.

Body black, clothed with greyish brown to pale brown scaling, sometimes mottled with small darker spots along elytral striae. Head with shallow, indistinct punctation concealed by scaling; eyes widely separated, interocular distance greater than interscrobal distance. Rostrum slightly longer than head, sides weakly narrowed anteriorly, dorsally nearly flat, with a shallow median impression with a fine carina extending onto frons. Antennae with scape only slightly exceeding anterior margin of prothorax; funicular antennomeres 1 and 2 subequal. Prothorax transverse; sides subparallel or slightly rounded in anterior half, then strongly constricted posteriorly; posterolateral angles acute; posterior margin moderately bisinuate, as wide as anterior margin; dorsal surface with large shallow punctures obscured by scaling and with a shallow transverse impression anterior to posterior margin. Elytra subparallel-sided; elytral striae narrow and finely punctate as visible through scaling; elytral interstriae almost flat, bearing very short, dense, sub depressed setae with circular scales. Legs dark brown; profemora bidentate, metafemora tridentate; tibiae with inner margin deeply emarginate proximally, especially metathoracic legs.

##### Remarks.

This species is extremely close to *M. discolor* Boheman, 1834 in general structure, but differs in the following characters: rostrum very slightly narrow anteriorly, slightly longer than head; prothorax transverse, posterior margin moderately bisinuate; elytral striae narrow, finely punctate as visible through scaling. Also, it is similar to *M. sabulosus* Marshall, 1916 in general appearance but easily distinguished, by having only one tooth on metafemora instead of three (Marshall 1916).

##### Plant association.

*Zea mays* (Poaceae), *Pennisetum glaucum* (Poaceae), *Sorghum bicolor* (Poaceae), *Setaria italica* (Poaceae), *Gossypium* spp. (Malvaceae), *Vigna mungo* (Fabaceae), *Glycine max* (Fabaceae), *Vigna unguiculata* (Fabaceae), *Trifolium alexandrinum* (Fabaceae), *Cyamopsis tetragonoloba* (Fabaceae), *Abelmoschus esculentus* (Malvaceae), *Luffa acutangula* (Cucurbitaceae), *Prunus dulcis* (Rosaceae), *Crotalaria juncea* (Fabaceae), *Alcea rosea* (Malvaceae), *Rosa* spp. (Rosaceae); attracted to light (Ramamurthy and Ghai 1988).

##### Distribution.

Sri Lanka, Afghanistan, India.

#### Myllocerus discolor

Taxon classificationAnimaliaColeopteraCurculionidae

Boheman, 1834

D375FCE4-A567-5DA3-B871-92B85AFF560A

Fig. 3C, D

Myllocerus discolor Boheman, 1834: 428. Type locality: Bengal; Type repository: Swedish Museum of Natural History, Stockholm. Type not examined.
= Myllocerus variegatus Boheman, 1842: 9; Marshall 1916: 348; Ramamurthy and Ghai 1988: 459. Type locality: Bengal; Type repository: Swedish Museum of Natural History, Stockholm.
= Myllocerus uniformis Marshall, 1916: 349; Ramamurthy and Ghai 1988: 459. Type locality: India-Mundali, Jaunsar; Type repository: NHMUK.
= Myllocerus canescens Marshall, 1916: 350; Ramamurthy and Ghai 1988: 459. Type locality: Ceylon-Halupahani; Type repository: NHMUK.
= Myllocerus persicus Zumpt, 1938: 84; Ramamurthy and Ghai 1988: 459. Type locality: Japan; Type repository: Natural History Museum in Basel, Switzerland.

##### Material examined.

Sri Lanka • Uva Province, Halupahani, Haldummulle; NHMUK, NHMUK013663894.

##### Diagnosis.

Integument black, densely covered with greyish white scales, rendering elytra almost entirely of this color, except for a faint, narrow pale-brown stripe extending anteriorly from elytral humeri. Head and broad lateral stripes of the prothorax clothed with pale-brown scales. Eyes widely separated and convex. Rostrum slightly widened apically; posterior interocular distance greater than interscrobal distance. Antennae with scape only slightly exceeding anterior margin of prothorax, funicular antennomeres 1 and 2 subequal. Prothorax weakly transverse, nearly as long as broad, with posterior margin deeply bisinuate. Elytral striae broad, with coarse punctures; setae markedly longer and more conspicuous on the disc. Legs robust; metafemora distinctly tridentate.

##### Remarks.

For comparison with *M. dentifer* (Fabricius, 1792), see above.

##### Plant association.

*Triticum aestivum* (Poaceae), *Oryza sativa* (Poaceae), *Zea mays* (Poaceae), *Sorghum bicolor* (Poaceae), *Pennisetum glaucum* (Poaceae), *Eleusine coracana* (Poaceae), *Gossypium* spp. (Malvaceae), *Saccharum officinarum* (Poaceae), *Corchorus* spp. (Malvaceae), *Vigna aconitifolia* (Fabaceae), *Ipomoea batatas* (Convolvulaceae), *Mangifera indica* (Anacardiaceae), *Ficus carica* (Moraceae), *Ziziphus mauritiana* (Rhamnaceae), *Musa* spp. (Musaceae), *Litchi chinensis* (Sapindaceae), *Aegle marmelos* (Rutaceae), *Citrus limon* (Rutaceae), *Psidium guajava* (Myrtaceae), *Eriobotrya japonica* (Rosaceae), *Morus* spp. (Moraceae), *Abelmoschus esculentus* (Malvaceae), *Cucurbita* spp. (Cucurbitaceae), *Moringa oleifera* (Moringaceae), *Medicago sativa* (Fabaceae), *Tectona grandis* (Lamiaceae), *Sesbania bispinosa* (Fabaceae), *Sesbania* spp. (Fabaceae), *Vachellia nilotica* (= *Acacia intsia*) (Fabaceae), *Dalbergia sissoo* and *Dalbergia paniculata* (Fabaceae), *Ziziphus jujuba* (Rhamnaceae), *Citrus* spp. (Rutaceae), *Solanum melongena* (Solanaceae), *Glycine max* (Fabaceae) (Marshall 1916; Ramamurthy and Ghai 1988).

##### Distribution.

Sri Lanka, India, Iran, Pakistan.

#### Myllocerus equinus

Taxon classificationAnimaliaColeopteraCurculionidae

Ramamurthy & Ghai, 1988

327F0DA6-221A-5131-8EDD-87EC8AEC8C47

Myllocerus equinus Ramamurthy & Ghai, 1988: 464. Type locality: Sri Lanka; Type repository: State Museum of Zoology, Dresden. Type not examined.

##### Material examined.

No specimens examined; data based on published records.

##### Diagnosis.

Body dark brown to black, densely clothed with dull white scales; elytra dull whitish, each bearing nine irregularly shaped dark brown-black spots. Head with median area densely covered with white scales; eyes lateral, interocular distance shorter than length of eye and nearly equal to interscrobal distance. Rostrum slightly longer than head, apically deeply and acutely emarginate; median carina distinct and well developed. Antennae with scape stout, barely surpassing anterior margin of prothorax; funicular antennomere 2 slightly longer than 1, funicular antennomere 3 longer than 4. Prothorax transverse, sides weakly narrowing anteriorly, strongly constricted posteriorly; posterior margin distinctly bisinuate and broader than anterior margin. Elytra with striae originating from base; strial punctures deep, oblong, and well defined; elytral interstriae bearing distinct scaly vestiture with opaque ridges, imparting a ribbed appearance, lateral elytral interstriae clearly demarcated. Legs dark brown with dull white scaling; profemora bidentate, metafemora tridentate (Ramamurthy and Ghai 1988).

##### Remarks.

This species differs from *M. undecimpustulatus undatus* Marshal, 1916 in elytra with strial punctures originating from base, punctures deep and oblong; elytral interstriae bearing distinct scaly vestiture with opaque ridges, imparting a ribbed appearance, lateral interstriae clearly demarcated; prothorax weakly narrowed anteriorly; elytral humeri not projecting laterally; posterior interocular distance nearly equal to interscrobal distance (Ramamurthy and Ghai 1988).

##### Plant association.

Unknown.

##### Distribution.

Sri Lanka.

#### Myllocerus fringilla

Taxon classificationAnimaliaColeopteraCurculionidae

Faust, 1897

ED9F92A2-80DA-5B4B-985A-CF176487B58A

Myllocerus fringilla Faust, 1897: 356. Type locality: Malacca; Type repository: State Museum of Zoology, Dresden. Type not examined.

##### Material examined.

No specimens examined; data based on published records.

##### Diagnosis.

Antennal scape not reaching middle of pronotum. Long rostrum with a distinct median impression, sometimes with a fine glabrous median line extending onto frons. Ventral surface distinctly and darkly punctate, similar to dorsal surface. Antennae and legs vary in color, ranging from yellowish red to dark brown. Pronotum and elytra more distinctly punctate; elytral strial punctures becoming larger and deeper towards base, elytral interstriae weakly convex; elytral apex strongly beak-shaped produced downward; femora bidentate (Faust 1897; Marshall 1916).

##### Remarks.

This species is closely related to *M. lineatocollis* (Boheman, 1842), but differs by the following characters: rostrum being longer with a distinct impression; elytra produced downwards at apex; having two femoral teeth (Marshall 1916).

Ramamurthy and Ghai (1988) listed *Myllocerus fringilla* Faust, 1897 as a synonym of M. lineatocollis var. frontalis Faust, 1897, citing Faust (1897: 363). However, the original description by Faust (1897) established only *M. frontalis* Faust, 1897, and not a form under *M. lineatocollis*; the latter thus appears to be a subsequent misinterpretation. *Myllocerus lineatocollis* (Boheman, 1842) is currently treated as a valid species, with *M. frontalis* as its synonym. Comparison of the original descriptions indicates that *M. fringilla* is distinct, particularly in the structure of the rostrum and the form of the elytral apex. Therefore, the treatment by Wijesinghe and Hong (2025), which synonymized M. lineatocollis var. frontalis with *M. fringilla*, is based on an invalid name and is considered erroneous.

##### Plant association.

Unknown.

##### Distribution.

Sri Lanka, Malacca.

#### Myllocerus subfasciatus

Taxon classificationAnimaliaColeopteraCurculionidae

Guérin-Méneville, 1843

9C159207-9F95-5762-9F39-75ACEF2D1653

Fig. 4A, B

Myllocerus subfasciatus Guérin-Méneville, 1843: 54. Type locality: India-Neelgheries; Type repository: Swedish Museum of Natural History, Stockholm. Type not examined.
= Myllocerus spurcatus Walker, 1859: 263; Marshall 1916: 345; Ramamurthy and Ghai 1988: 456. Type locality: Ceylon; Type repository: NHMUK.

##### Material examined.

Sri Lanka • 1♂, Western Province, Weliweriya, 7°1.96'N, 80°1.6'E; 25 Jul. 2023; KJ. Hong leg.; on *Solanum melongena* (Solanaceae); SCNU • 1♂; Central Province, Dikoya, 3800–4200 ft; 6 Dec. 1881–16 Jan. 1882; G. Lewis leg.; NHMUK, NHMUK013663889.

**Figure 4.**
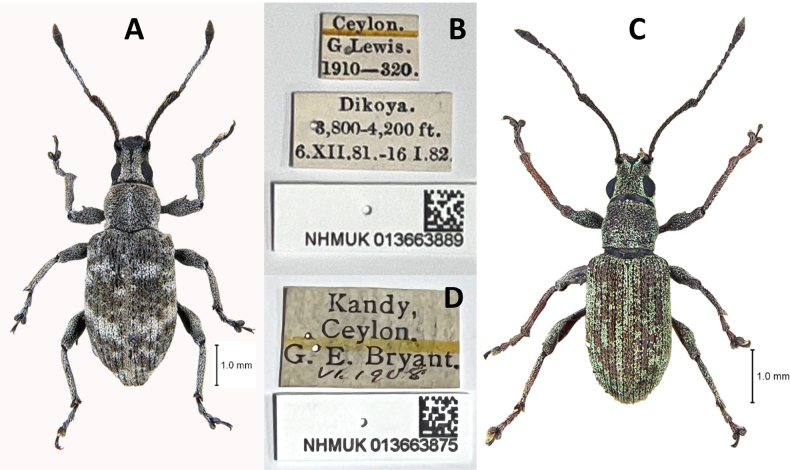
Sri Lankan *Myllocerus* Schönherr, 1823 species examined from National History Museum, London. **A, B**. *Myllocerus subfasciatus* Guérin-Méneville, 1843; **A**. Habitus; **B**. Labels; **C, D**. *Myllocerus zeylanicus* Marshall, 1916; **C**. Habitus; **D**. Labels.

##### Diagnosis.

Body black, clothed with coarse brown scaling; prothorax usually paler laterally and bearing a paler median line; elytra variably irrorated with greyish or whitish scales, typically forming faint transverse bands, occasionally more distinct and conspicuous. Head with a distinct median fovea; eyes lateral; interocular distance broader than eye width and slightly broader than interscrobal distance. Rostrum slightly longer than head, weakly dilated apically, dorsally rather deeply impressed, with a narrow smooth median line extending onto frons. Antennae stout; scape bearing dense suberect setae; funicular antennomere 2 longer than 1, funicular antennomeres 3 and 4 subequal; funicular antennomeres 2–6 with conspicuous white setae. Prothorax transverse, sides strongly rounded, deeply constricted and transversely impressed posteriorly, more shallowly constricted anteriorly; posterior margin distinctly bisinuate; a shallow rounded lateral impression present posterior to mid-length. Elytra relatively broad, elytral humeri rectangularly rounded; elytral striae broad, deeply punctate; vestiture composed of dense, short, sub depressed, squamiform setae. Legs dark brown with dense grey or brown scaling; femora bearing two unequal teeth; inner margin of tibia with a slight angular projection.

##### Remarks.

For comparison with *M. curvicornis* (Fabricius, 1792), see above.

##### Plant association.

*Gossypium* spp. (Malvaceae), *Corchorus* spp. (Malvaceae), *Arachis hypogaea* (Fabaceae), *Solanum melongena* (Solanaceae), *Solanum tuberosum* (Solanaceae), *Malus domestica* (Rosaceae), *Prunus* spp. (Rosaceae), *Boehmeria nivea* (Urticaceae), *Alcea rosea* (Malvaceae); attracted to light (Ramamurthy and Ghai 1988; Shanmugam et al. 2018).

##### Distribution.

Sri Lanka, India, Myanmar.

#### Myllocerus undecimpustulatus undatus

Taxon classificationAnimaliaColeopteraCurculionidae

Marshall, 1916

DA3F2FAC-0EEC-5F1D-B7B4-BD665A2334D5

Fig. 5A–D

Myllocerus undatus Marshall, 1916: 350.
Myllocerus undecimpustulatus undatus : O’Brien et al. 2006: 1. Type locality: Ceylon-Weligama; Type repository: NHMUK. Type not examined.

##### Material examined.

Sri Lanka • 1♀, Central Province, Kandy; Jun. 1905; GE. Bryant leg.; NHMUK, NHMUK013663888; • 1♂, Western Province, Colombo; Sep. 1890; “HP. Green” [EE. Green] leg.; NHMUK, NHMUK013663891.

**Figure 5.**
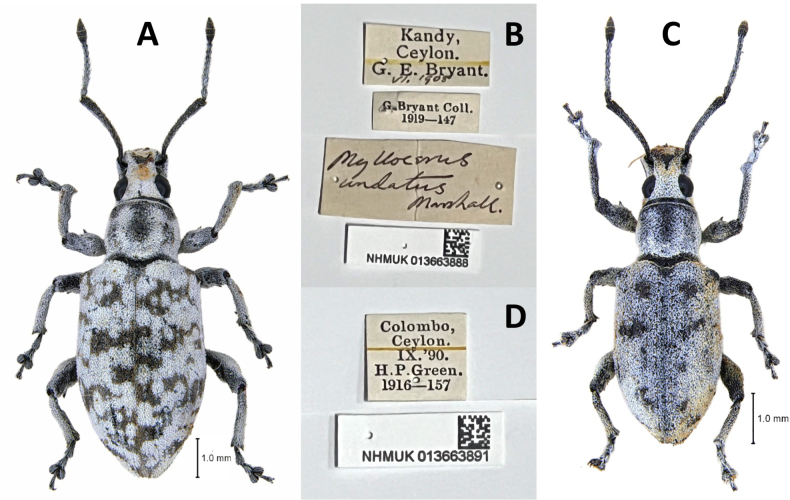
*Myllocerus undecimpustulatus undatus* Marshall, 1916 examined from National History Museum, London. **A, B**. Specimen collected from Kandy in Sri Lanka. **A**. Habitus; **B**. Labels; **C, D**. Specimen collected from Colombo in Sri Lanka. **C**. Habitus; **D**. Labels.

##### Diagnosis.

Body maculate, clothed with dull white and black scales, often with yellowish scales on rostrum and head; prothorax bearing three darker, partially denuded longitudinal stripes, lateral stripes interrupted; elytra with more or less distinct large dark brown blotches, usually arranged into three irregular oblique bands. Eyes sublateral; posterior interocular distance greater than interscrobal distance. Antennae stout; scape with dense suberect setae; funicular antennomere 2 longer than 1, funicular antennomeres 3 and 4 subequal. Rostrum slightly longer than head, weakly dilated apically, with a narrow smooth median line confined to posteriorly. Prothorax weakly rounded laterally, constricted posteriorly; posterior margin bisinuate. Elytral humeri strongly angulate; projecting laterally and distinctly broader than prothorax. Legs with profemora and mesofemora bidentate, metafemora distinctly tridentate.

##### Remarks.

For comparison with *M. equinus* Ramamurthy & Ghai, 1988, see above. This subspecies is closely related to *Myllocerus undecimpustulatus undecimpustulatus* Faust, 1891, but can be distinguished primarily by its narrower interocular distance, which is not broader than the length of an eye.

##### Plant association.

*Oryza sativa* (Poaceae), *Zea mays* (Poaceae), *Cajanus cajan* (Fabaceae), *Gossypium* spp. (Malvaceae), *Corchorus* spp. (Malvaceae), *Helianthus annuus* (Asteraceae), *Mangifera indica* (Anacardiaceae), *Punica granatum* (Lythraceae), *Ziziphus mauritiana* (Rhamnaceae), *Fragaria × ananassa* (Rosaceae), *Malus domestica* (Rosaceae), *Medicago sativa* (Fabaceae), *Sesbania bispinosa* (Fabaceae), *Dalbergia sissoo* (Fabaceae), *Imperata cylindrica* (Poaceae), *Psophocarpus tetragonolobus* (Fabaceae), *Citrus* spp. (Rutaceae), *Solanum melongena* (Solanaceae), *Daucus carota* (Apiaceae), *Ipomoea batatas* (Convolvulaceae), *Conocarpus erectus* (Combretaceae), *Bauhinia × blakeana* (Fabaceae), *Chrysobalanus icaco* (Chrysobalanaceae), *Phoenix roebelenii* (Arecaceae), *Prunus persica* (Rosaceae), *Lagerstroemia indica* (Lythraceae), *Capsicum* spp. (Solanaceae), *Litchi chinensis* (Sapindaceae), *Muntingia calabura* (Muntingiaceae), *Fragaria × ananassa* (Rosaceae) and *Solanum melongena* (Solanaceae) (Butani 1979; Ramamurthy and Ghai 1988; Neal 2013; Rajan and Ghosh 2019).

##### Distribution.

Sri Lanka, adventive in the Southeastern United States (Hunsberger 2003; O’Brien et al. 2006).

#### Myllocerus viridanus

Taxon classificationAnimaliaColeopteraCurculionidae

(Fabricius, 1775)

DA91AA60-5926-598E-8E3E-B1BAA6C981DB

Curculio viridanus Fabricius, 1775: 155. Type locality: India-Tranquebar; Type repository: Zoological Museum, University of Copenhagen. Type not examined.
= Myllocerus angustifrons Faust, 1897: 356; Marshall 1916: 301; Ramamurthy and Ghai 1988: 414. Type locality: India; Type repository: State Museum of Zoology, Dresden.

##### Material examined.

No specimens examined; data based on published records.

##### Diagnosis.

Body covered with dense, uniform light-green scales, sometimes varying to pale greenish white; in paler specimens a chalky-white efflorescence may be present. Head tinged with yellow, bearing metallic green scales at apex of rostrum; narrowed anteriorly, with straight sides. Eyes dorsal causing external margins of both eyes plainly visible from above at same time and converging slightly anteriorly, frons with a median, longitudinal impression. Rostrum evidently longer, broader than head; sides straight; scrobes entirely dorsal, interscrobal distance slightly broader than interocular distance. A low, rounded longitudinal ridge originates from anteroventral margin of eye and curves indistinctly inward near mid-length. Antennae with funicular antennomeres 1 and 2 elongate and subequal; funicular antennomere 3 slightly longer than 4, and funicular antennomere 4 longer than 5. Prothorax subconical, widest posteriorly and strongly narrowed anteriorly; sides straight; posterior margin deeply bisinuate; dorsal surface bearing deep, well-separated punctures concealed by scaling. Elytra almost parallel-sided from elytral humeri to posterior to mid-length in ♂, gradually rounded toward the apex and with laterally prominent elytral humeri in ♀; vestiture with scales on elytral interstriae more oval than circular. Legs black with green scaling; femora bidentate, outer tooth consistently small (Marshall 1916).

##### Remarks.

For comparison with *M. delicatulus* Boheman, 1842, see above.

##### Plant association.

*Oryza sativa* (Poaceae), *Corchorus* spp. (Malvaceae), *Arachis hypogaea* (Fabaceae), *Ricinus communis* (Euphorbiaceae), *Linum usitatissimum* (Linaceae), *Helianthus annuus* (Asteraceae), *Ipomoea batatas* (Convolvulaceae), *Trichosanthes cucumerina* (Cucurbitaceae), *Lablab purpureus* (Fabaceae), *Abelmoschus esculentus* (Malvaceae), *Citrus limon* (Rutaceae), *Mangifera indica* (Anacardiaceae), *Psidium guajava* (Myrtaceae), *Carica papaya* (Caricaceae), *Manilkara zapota* (Sapotaceae), *Ficus carica* (Moraceae), *Prunus persica* (Rosaceae), *Theobroma cacao* (Malvaceae), *Camellia sinensis* (Theaceae), *Rosa* spp. (Rosaceae), *Indigofera* spp. (Fabaceae), *Plumbago zeylanica* (Plumbaginaceae), *Sesbania* spp. (Fabaceae), *Tectona grandis* (Lamiaceae), *Eucalyptus* spp. (Myrtaceae), *Acalypha* spp. (Euphorbiaceae), *Lantana camara* (Verbenaceae); attracted to light (Ramamurthy and Ghai 1988).

##### Distribution.

Sri Lanka, India.

#### Myllocerus zeylanicus

Taxon classificationAnimaliaColeopteraCurculionidae

Marshall, 1916

FB66E058-1A61-5844-8095-B11A8FFB8920

Fig. 4C, D

Myllocerus zeylanicus Marshall, 1916: 315. Type locality: Ceylon-Kandy; Type repository: NHMUK. Type not examined.

##### Material examined.

Sri Lanka • 1♂, Central Province, Kandy; 1550–1700 ft; Jun. 1908; GE. Bryant leg.; NHMUK, NHMUK013663875.

##### Diagnosis.

Body dark reddish brown, clothed with grey to pale grey-green scales, very sparse dorsally but much denser on lateral surfaces and ventrally. Head minutely striolate beneath scaling; eyes large and lateral; interocular distance nearly as broad as interscrobal distance. Rostrum slightly longer than head and nearly as long as broad; sides narrowed from base to mid-length and slightly dilated anteriorly; apical emargination moderately deep, forming an acute angle. Dorsal surface of rostrum shallowly impressed, with faint lateral carinae and a very fine median carina extending onto frons. Antennae dark reddish brown; funicular antennomeres 1 and 2 equal in length, funicular antennomere 3 distinctly longer than 4; funicular antennomere 7 longer than broad; club elongate, club antennomere 1 conical, as long as broad and not shorter than 2. Prothorax about as long as broad, with sides slightly rounded; shallowly constricted and transversely impressed near both posterior and anterior margins; posterior margin subtruncate; anterior margin of uniform width, oblique laterally; dorsal surface densely punctate. Elytra subtruncate at posterior margin, parallel-sided in ♂ and broader posteriorly in ♀; dorsally almost flat; elytral humeri roundly rectangular; elytral striae deeply punctate; elytral interstriae bearing short, suberect, pale, curved setae. Legs dark reddish brown; femora with a sharp tooth.

##### Remarks.

This species is closely related to *M. angulatipes* Marshall, 1916 see above. Also, it is closely related to *M. hispidus* Marshall, 1916, but differs by the following characters: rostral apex with apical emargination moderately deep, forming an acute angle; funicular antennomere 7 longer than broad; antennal club elongate, with club antennomere 1 conical, as long as broad and not shorter than 2; and elytral interstriae bearing short, suberect, pale, curved setae, whereas in *M. hispidus* setae are longer, erect, and black (Marshall 1916).

##### Plant association.

Unknown.

##### Distribution.

Sri Lanka.

## Discussion

According to Ramamurthy and Ghai (1988) and Wijesinghe and Hong (2025), there are 11 species of *Myllocerus* recorded from Sri Lanka. In this review, we examined 7 species from NHMUK. *Myllocerus angulatipes*, *M. equinus* and *M. zeylanicus* can be considered endemic to the country. *Myllocerus curvicornis* and *M. undatus* are currently considered the most widespread native species in Sri Lanka (Wijerathna et al. 2024).

*M. undatus* was originally described by Marshall (1916) as a distinct species based primarily on scaling patterns, without a detailed discussion of additional diagnostic morphological characters. In the original description, Marshall differentiated *M. undatus* from *M. undecimpustulatus* mainly by the interocular distance compared with the length of eye, whereas in the illustrated key, he used the relative interscrobal and interocular distances and the position of the elytral humeri angle. This interpretation of the illustrated key was subsequently followed by Ramamurthy and Ghai (1988) without critically re-evaluating *M. undatus*. Subsequently, O’Brien et al. (2006) treated the species as *M. undecimpustulatus undatus*, although without explicit justification. Their study demonstrated variation in scaling patterns among adventive populations identified as *M. undecimpustulatus undatus* in Florida, suggesting that these characters may be variable within populations and insufficient alone for species-level separation. In addition, O’Brien et al. (2006) reported broad host associations and strong dispersal ability in adventive populations, indicating that the species represents a widespread and ecologically adaptable lineage. The present study tentatively follows the subspecies interpretation of O’Brien et al. (2006), as the diagnostic characters proposed by Marshall (1916) appear insufficient to support separate species status and may instead reflect geographic variation within the morphologically variable *M. undecimpustulatus* lineage. Also, *M. undecimpustulatus* is native to southern India (O’Brien et al. 2006), whereas *M. undatus* is native to Sri Lanka (Wijerathna et al. 2024). Considering the close biogeographic relationship between southern India and Sri Lanka, together with the geographic isolation of Sri Lankan populations, the Sri Lankan species may represent an insular geographic form derived from the southern Indian lineage rather than a fully distinct species. Nevertheless, the geographic isolation of the Sri Lankan population and the persistence of minor but consistent morphological differences argue against its complete synonymization with *M. undecimpustulatus* at present. However, comprehensive comparative morphological and molecular analyses across Indian and Sri Lankan populations are still lacking; therefore, the taxon is provisionally retained as *M. undecimpustulatus undatus* pending further study.

**Figure 6.**
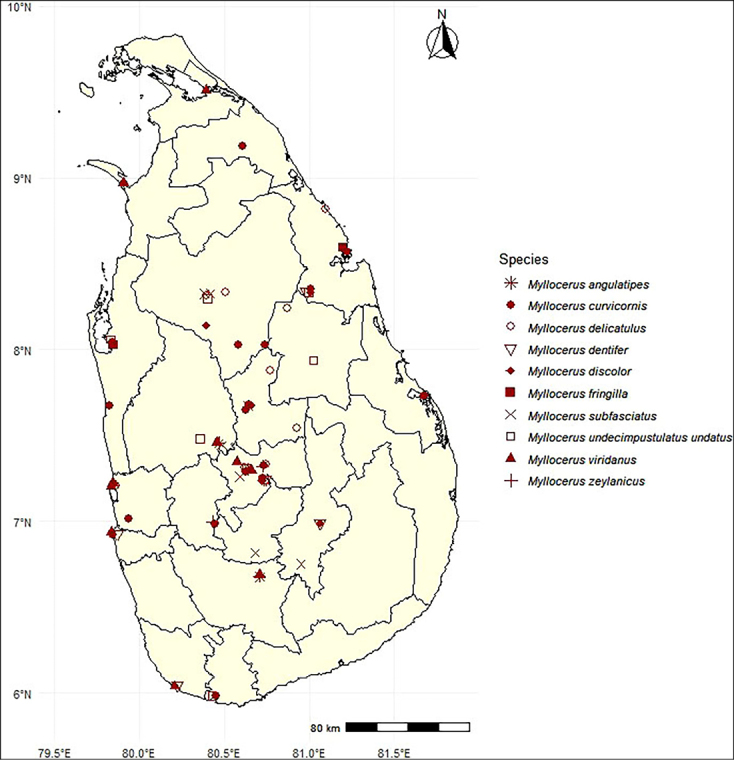
*Myllocerus* Schönherr, 1823 species recorded from Sri Lanka by Districts.

**Figure 7.**
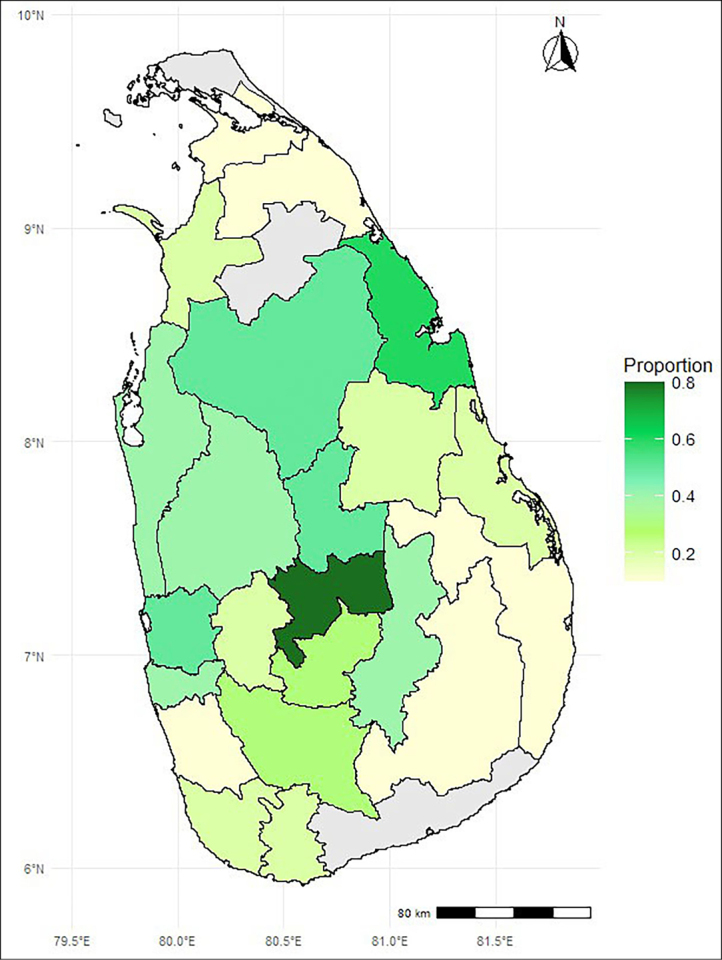
Proportional species richness among districts relative to the total *Myllocerus* Schönherr, 1823 diversity of Sri Lanka.

Species of the genus *Myllocerus* have been recorded from 22 of 25 Districts of Sri Lanka (Figs 6, 7): Ampara (2 species), Anuradhapura (5 species), Badulla (4 species), Batticaloa (2 species), Colombo (4 species), Galle (2 species), Gampaha (4 species), Kalutara (1 species), Kandy (8 species), Kegalle (2 species), Kilinochchi (1 species), Kurunegala (4 species), Mannar (2 species), Matale (5 species), Matara (2 species), Monaragala (1 species), Mullativu (1 species), Nuwara Eliya (3 species), Polonnaruwa (1 species), Puttalam (4 species), Ratnapura (3 species) and Trincomalee (6 species). Therefore, most records are known from Kandy District (Fig. 7), followed by Trincomalee, Matale and Anuradhapura Districts. Compared to the neighboring country, the number of recorded species in Sri Lanka is relatively low. In India, 90 species have been recorded (Ramamurthy and Ghai 1988; Nagaraja 2019). However, the limited number of known species in Sri Lanka is more indicative of inadequate targeted research than of the true diversity of the group in the country.

**Figure 8.**
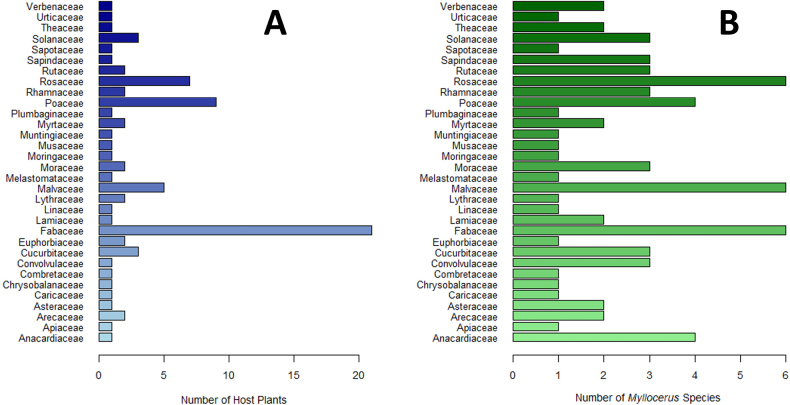
Host plant family associations of *Myllocerus* Schönherr, 1823 species. **A**. Number of recorded host plants within each plant family; **B**. Number of *Myllocerus* species associated with different host plant families.

**Figure 9.**
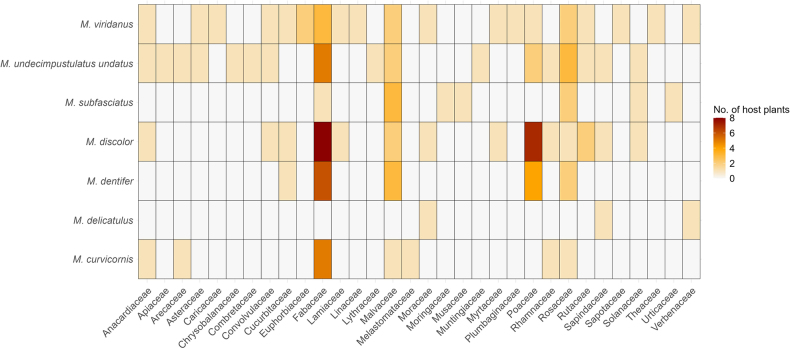
Heatmap of *Myllocerus*–host plant interactions aggregated by plant family.

The host plant range of *Myllocerus* species is also notably broad. Based on available records, they are associated with more than 80 host plants from 32 families (Fig. 8A). Among these, Fabaceae (21 taxa), Poaceae (9 taxa), Rosaceae (7 taxa), and Malvaceae (5 taxa) represent the most important families in terms of host plant diversity, and they also support a relatively high number of associated *Myllocerus* (6, 4, 6, and 6 species, respectively) (Figs 8B, 9). The polyphagous diet of *Myllocerus* is especially highlighted by broad spectra of associated plant families, particularly *M. viridanus* (19 families), *M. undecimpustulatus undatus* (17 families), and *M. discolor* (16 families) (Fig. 9). Such extensive host associations indicate a high degree of ecological adaptability and contribute to their potential importance as agricultural pests. The diversity of host plants further emphasizes the economic relevance of this genus in Sri Lanka, where several crops are affected.

Observations from iNaturalist citizen science platform and GBIF database have revealed *M. paetus* and *M. dorsatus*, which have not been previously recorded in the literature. These observations suggest that additional species may occur in Sri Lanka. However, specimen collection, careful examination, and the designation of voucher specimens are necessary to confirm new country records. The number of recorded species is expected to increase once collection-based research and targeted field studies on Entiminae are conducted.

Among known pests in Sri Lanka, *M. undecimpustulatus undatus* and *M. viridanus* have been reported to damage a wide range of crops through characteristic leaf notching, defoliation, and root feeding (Butani 1979; Shanthichandra et al. 1990; Neal 2013; Rajan and Ghosh 2019). Other species, including *M. discolor* and *M. subfasciatus*, also contribute to crop damage (Shanmugam et al. 2018). Therefore, accurate species-level identification is of practical importance for agriculture in Sri Lanka. Reliable identification helps farmers, extension officers, and pest management experts to determine which species are dealing with. This enables them to evaluate possible damage and to choose suitable control measures. Incorrect identification may result in ineffective or unnecessary pesticide use, increasing both costs and environmental impacts. For this reason, documenting the species diversity and diagnostic characters of *Myllocerus* in Sri Lanka contributes to both taxonomic knowledge and to sustainable pest management practices.

Curculionids are scarce in Sri Lankan entomological collections. Identified specimens are generally rare, largely due to the lack of national taxonomic resources for most weevil groups. Digitized records are also limited and often identified only at the family level. Extensive taxonomic work, biodiversity surveys, including revisions of most genera, are necessary to enable accurate synoptic collections. Resources such as the present study are important for improving the availability of taxonomic tools for researchers in Sri Lanka. In addition, facilitating access to preserved specimens and images through online databases, e.g. GBIF would help to better document local diversity and support future identifications. The establishment and strengthening of local voucher collections are therefore strongly recommended. The specimens collected and identified in the present study could contribute to such efforts, and future work should prioritize the deposition of well-curated material in local institutions.

## Supplementary Material

XML Treatment for Myllocerina

XML Treatment for Myllocerus

XML Treatment for Myllocerus angulatipes

XML Treatment for Myllocerus curvicornis

XML Treatment for Myllocerus delicatulus

XML Treatment for Myllocerus dentifer

XML Treatment for Myllocerus discolor

XML Treatment for Myllocerus equinus

XML Treatment for Myllocerus fringilla

XML Treatment for Myllocerus subfasciatus

XML Treatment for Myllocerus undecimpustulatus undatus

XML Treatment for Myllocerus viridanus

XML Treatment for Myllocerus zeylanicus

## Additional information

### Artificial Intelligence (AI) use

The authors accept full responsibility for the content of the manuscript, including the disclosure of any use of AI.

No AI tools were used in the preparation of this manuscript.

### Author ORCIDs

D.D. Wijesinghe https://orcid.org/0009-0005-1630-6770

K.-J. Hong https://orcid.org/0000-0001-7002-6976

### Data availability

All of the data that support the findings of this study are available in the main text or Supplementary Information.

## Supplementary materials

Supplementary material 1 Geographic locality coordinates of *Myllocerus* species recorded from Sri Lanka Data type xlsx https://binary.pensoft.net/file/1706126This dataset is made available under the Open Database License (http://opendatacommons.org/licenses/odbl/1.0/). The Open Database License (ODbL) is a license agreement intended to allow users to freely share, modify, and use this Dataset while maintaining this same freedom for others, provided that the original source and author(s) are credited. Dilshara D. Wijesinghe, Ki-Jeong Hong
